# pH-mediated control over the mesostructure of ordered mesoporous materials templated by polyion complex micelles

**DOI:** 10.3762/bjnano.10.14

**Published:** 2019-01-11

**Authors:** Emilie Molina, Mélody Mathonnat, Jason Richard, Patrick Lacroix-Desmazes, Martin In, Philippe Dieudonné, Thomas Cacciaguerra, Corine Gérardin, Nathalie Marcotte

**Affiliations:** 1 ICGM UMR 5253 CNRS – Université de Montpellier - ENSCM, ENSCM 240 Av Pr E. Jeanbrau, 34296 Montpellier cedex 5, France,; 2 Laboratoire Charles Coulomb, UMR 5221 CNRS – Université de Montpellier, 34095 Montpellier, France

**Keywords:** double-hydrophilic block copolymer, hybrid organic–inorganic interface, mesoporous materials, nanostructured materials, polyion complex micelles, polyion electrostatic complexation

## Abstract

Ordered mesoporous silica materials were prepared under different pH conditions by using a silicon alkoxide as a silica source and polyion complex (PIC) micelles as the structure-directing agents. PIC micelles were formed by complexation between a weak polyacid-containing double-hydrophilic block copolymer, poly(ethylene oxide)-*b*-poly(acrylic acid) (PEO-*b*-PAA), and a weak polybase, oligochitosan-type polyamine. As both the micellization process and the rate of silica condensation are highly dependent on pH, the properties of silica mesostructures can be modulated by changing the pH of the reaction medium. Varying the materials synthesis pH from 4.5 to 7.9 led to 2D-hexagonal, wormlike or lamellar mesostructures, with a varying degree of order. The chemical composition of the as-synthesized hybrid organic/inorganic materials was also found to vary with pH. The structure variations were discussed based on the extent of electrostatic complexing bonds between acrylate and amino functions and on the silica condensation rate as a function of pH.

## Introduction

Due to their unique physicochemical properties originating from their uniform pore size and periodically arranged network at the mesoscale, silica-based ordered mesoporous materials (OMMs) have attracted considerable attention in various fields such as adsorption, separation and catalysis. The formation of these mesostructures relies on a supramolecular assembly process between silicic species and surfactants or amphiphilic block copolymers acting as structure directing agents (SDAs) of silica. The assembly process can occur following two different interaction pathways: one is based on an electrostatic charge-matching mechanism between the SDA (cationic S^+^, anionic S^−^ or protonated neutral S^0^ denoted as S^0^H^+^) and inorganic species (I^+^ or I^−^) interacting either directly or through a mediator species (halide anion X^−^ or alkaline cation M^+^),[1] while the other one proceeds through an electrically neutral route involving hydrogen bond interactions between neutral amine (S^0^) [2] or poly(ethylene oxide) (N^0^) [3] based SDA and neutral inorganic species (I^0^). Concerning the poly(ethylene oxide) based SDA, it is believed that the nature of the attractive interaction is hydrogen bonding between the silanol groups and the ether oxygen in the PEO block [4–5], as it is the case when PEO adsorbs on a silica surface [6]. It should be noted that the charge-matching pathway requires extreme pH conditions to produce OMMs, as for the synthesis of the well-known SBA (Santa Barbara Amorphous) and M41S (from Mobil Corporation) materials families, which proceed at pH < 1 and pH > 9, respectively. On the contrary, the neutral route necessitates less severe pH conditions, which are much more appropriate for a large-scale material production with controlled environmental, health and safety risks. Among the SDA materials that have been employed, non-ionic block copolymers such as poly(alkylene oxide) triblock copolymers have attracted more attention due to the formation of thermally and mechanically stable materials with larger pore sizes and thicker walls than those obtained with surfactants. This opens up the possibility of easily functionalizing them and tailoring the mesopore arrangement into various ordered mesostructures [7]. This is especially true as long as the material synthesis is conducted under strongly acidic conditions (charge-matching pathway (S^0^H^+^)(X^−^I^+^)) [4,8]. In contrast, the first attempts to synthesize materials in quasi neutral solution (pH > 2) using non-ionic block copolymers, which involve weaker interactions between the PEO chains and neutral silicic species, resulted in more disordered framework structures with worm-like mesopore channels of uniform diameter [3], designed as MSU-*X*. It should be mentioned that the reported synthesis protocols made use of synchronous reaction steps involving co-assembly of the template and the inorganic precursor and hydrolysis and condensation of the silica precursor. Bearing this in mind, the lack of a well-defined periodic structure of MSU-*X* may be imputed to the type of silica precursor generally used, namely alkoxysilanes like tetraethoxysilane (TEOS), whose rate of hydrolysis and condensation varies inversely as a function of pH [9]. Thus, synthesis at pH values above the isoelectric point of silica promotes condensation reactions that occur between silica species, which are only partially hydrolyzed, whereas fully hydrolyzed monomeric silica species would be desired for producing mesostructures with optimal structural order. As a matter of fact, using a rapid hydrolyzing alkoxysilane such as tetramethoxysilane (TMOS) that hydrolyses faster than TEOS, Kim et al. evidenced the possibility to produce hexagonally ordered structures from Pluronic P123 up to pH 4. The structural order can even be further extended up to pH 9 in the presence of fluoride ions due to their catalytic activity in hydrolysis reactions [10].

Several synthesis approaches aiming at separating the hydrolysis and the condensation steps were subsequently proposed to produce ordered mesostructured silica materials under mild pH conditions. Among them the use of sodium silicate as a silica source instead of silicon alkoxide judiciously discards the hydrolysis reactions. Adding silicate directly to a non-ionic block copolymer solution made of acid [11–12] or buffer [13–16] media allowed materials with ordered mesopores to be obtained up to pH 6.5. Interestingly, this relatively inexpensive silica precursor and the rather environmentally friendly synthesis route employed (neutral pH conditions, low temperature, short synthesis and aging times) open up new opportunities for batch and continuous mode large-scale production of ordered mesoporous silica materials [17–18]. Alternatively, the hydrolysis and condensation steps of the more popular silicon alkoxide precursors can be separated following a two-step approach in which the hydrolysis of the alkoxysilane is first performed in acidic medium and the silica condensation step is triggered with the aid of sodium fluoride and/or pH adjustment [19]. Separating the hydrolysis of TEOS from the condensation step enabled highly ordered 2D hexagonal SBA-15-type [20] and cubic [21–22] SBA-16-type materials to be obtained up to pH 5 and pH 4–4.5, respectively.

Since 2008, we have been developing an original route for the synthesis of ordered mesoporous materials based on the use of non-conventional structure-directing agents. Ordered mesoporous silica and organosilica materials were prepared under mild acidic conditions by using polyion complex (PIC) micelles as versatile pH-sensitive structure-directing agents [23–24]. This route relies on the use of a weak polyacid double hydrophilic block copolymer (DHBC) able to form polyion complex micelles upon interaction with a weak polybase. We reported that DHBCs such as poly(ethylene oxide)-*b*-poly(acrylic acid) (PEO-*b*-PAA) or poly(ethylene oxide)-*b*-poly(methacrylic acid) (PEO-*b*-PMAA) copolymers, are able to form polyion complex (PIC) micelles upon interaction with weak polybases such as oligochitosan (OC) [25], poly-L-lysine (PLL) [26–27] and aminoglycoside antibiotics [28]. PIC micelles present a core–corona structure, whose core is formed by electrostatic interactions between the two charged blocks (i.e., the PAA and the weak polybase) and the corona is constituted by the neutral PEO block of the DHBC, which ensures the steric stabilization of the assembly in water. In the presence of silica precursors, the hybrid organic–inorganic interface, which is necessary for directing the macroscopic precipitation of the hybrid material, can form through an interaction between the PEO neutral block and silicic species via the N^0^/I^0^ pathway, as in the case of SBA-type materials synthesized under acidic conditions, as long as no competing interactions involving silicic species exist. In a previous study, we had shown that when the strength of the organic–inorganic interaction is kept constant (by synthesizing hybrid materials at a fixed pH), the material mesostructure can be controlled by varying parameters that alter interactions between the different constituents of the system, such as the molar ratio between the complexing units, the molar ratio between ethylene oxide (EO) units and silica species, and the mass concentration of the reactants in the synthesis medium [26]. In the present paper, we investigate how a simple synthesis parameter, such as the pH of the reaction medium, which governs not only the extent of the polyion electrostatic complexation but also the silica condensation rate, influences the macrophase separation of the hybrid material and the nature of the mesostructures which are obtained. The variations of the mesostructures and the chemical composition of the corresponding hybrid materials as a function of pH are reported and discussed.

## Experimental

### Materials

Poly(ethylene oxide)-*b*-poly(acrylic acid) (PEO-*b*-PAA, *M*_PEO_ = 5000 g·mol^−1^, *M*_PAA_ = 1420 g·mol^−1^) was synthesized by atom transfer radical polymerization (ATRP) according to published procedures [29]. All reactions were carried out in the absence of air using standard Schlenk techniques and vacuum-line manipulation. All the chemicals used for the reaction (tert-butyl acrylate 98%, α-methoxy-ω-hydroxy-poly(ethylene oxide) with *M*_n_ = 5000 g·mol^−1^, CuBr 98%, 1,1,1,7,10,10-hexamethyltriethylenetetramine 97%, trifluoroacetic acid 99%, triethylamine 99%, 2-bromoisobutyryl bromide 98%, absolute ethanol, toluene 99.8%, THF 99.9%, acetone 99.5%, diethyl ether 99.5%, glacial acetic acid, dichloromethane, pentane, sodium chloride, DOWEX MSC-H resin, neutral alumina 50–200 µm) were purchased from Sigma-Aldrich and purified when necessary (α-methoxy-ω-hydroxy-poly(ethylene oxide), tert-butyl acrylate, toluene, THF, acetone); the solvents were dried and distilled by routine procedures. Oligochitosan lactate (OC, M < 5000 g·mol^−1^), tetraethoxysilane (TEOS), nitric acid (HNO_3_) and sodium hydroxide (NaOH) were purchased from Aldrich and used as received.

#### Chemical composition of the oligochitosan

A detailed characterization of the commercial oligochitosan (OC) was undertaken in order to determine the chemical composition of the repetitive unit necessary to fix the quantity of nitrogen per acrylic acid (N/AA ratio) used in the materials synthesis. The deacetylation degree (DD = 83 ± 5%) was determined by solid state ^15^N NMR using a Varian VNMRS 600 spectrometer operating at 5 kHz using cross-polarization magic-angle spinning conditions [30]. The DD was calculated from the integration of the amide (δ = 101 ppm) and amine (10 ppm) peaks using the following formula:

[1] $$\%\text{DD}=\frac{I_{\text{N-amine}}}{I_{\text{N-amine}}+I_{\text{N-amide}}}\;*100.$$

Note that a similar DD was obtained from liquid ^1^H NMR data recorded on a Bruker 400 MHz spectrometer using the method reported by Trombotto et al. [30].

The quantity of lactate/lactic acid present in the sample was calculated from the liquid ^1^H NMR spectrum recorded in D_2_O. The amount of water was deduced from elemental analysis. The molar composition of the repetitive unit constituting the oligochitosan was then: H-(C_6_H_11_O_4_N)_0.83_(C_8_H_13_O_5_N)_0.17_-OH, 1.31(C_3_H_6_O_3_), 0.09(H_2_O), where C_6_H_11_O_4_N represents the deacetylated unit, C_8_H_13_O_5_N the acetylated unit and C_3_H_6_O_3_ the lactate ion.

#### Preparation of mesostructured silica materials

Mesostructured hybrid silica materials were prepared following a one-pot synthesis approach. The concentration of OC and TEOS were fixed with respect to the number of acrylic acid (AA) and ethylene oxide (EO) units of the PEO-*b*-PAA, using a molar ratio of 0.8 nitrogen per AA (N/AA = 0.8) and 1 silicon per EO (EO/Si = 1). The final concentration of the reaction medium was set at 3.9 wt % of PEO-*b*-PAA, unless otherwise specified. Typically, TEOS (0.397 mL) was added into a homogeneous aqueous solution (2.0 mL) containing PEO-*b*-PAA (100 mg) and OC (77.5 mg) and the pH of the reaction medium was adjusted to 2 using HNO_3_ (2 mol·L^−1^). After completion of TEOS hydrolysis (about 40 min under vigorous magnetic stirring), the pH was rapidly increased to a fixed and well-defined value (ranging from 4 to 7.9) by adding a small amount of an aqueous NaOH solution (3 mol·L^−1^). This results in macroscopic precipitation, which occurs more or less rapidly according to the pH value (3 min at pH 5 and 0.5 min at pH 7.9). The mixture was stirred slowly for 24 additional hours at 30 °C. The precipitate was then recovered by centrifugation and air-dried at 40 °C for 48 hours. For characterization purposes, the as-synthesized hybrid materials were calcined under air flow from room temperature to 550 °C at a heating rate of 2 °C·min^−1^ and then maintained at 550 °C for 8 hours.

#### Characterization techniques

The formation of polyion complex (PIC) micelles as a function of pH (3 < pH < 10) was studied by dynamic light scattering (DLS) experiments at 25 ± 1°C using a Malvern 4800 spectrogoniometer (Malvern Instruments, UK) equipped with a 50 mW laser operating at 532 nm. The scattered light was collected at an angle of 90°. The static scattered light intensities were corrected from the pinhole size and normalized with respect to a Rayleigh scattered reference (toluene). The intensity autocorrelation function was fitted using the CONTIN algorithm for the determination of the volume-averaged hydrodynamic diameters (*D*_hv_); the polydispersity index (PDI) values were obtained from the cumulant method.

The chemical composition of the hybrid materials was calculated by combining thermogravimetric analysis (TGA), which allows quantifying the silica content, and N and C elemental analyses (EA), which allow determining the quantity of OC and PEO-*b*-PAA in the material. The results were expressed as N/AA and EO/Si ratios given with ±5% relative error. TGA was performed on a Perkin Elmer STA 6000 instrument at a heating rate of 10 °C·min^−1^ under an air flow (20 mL·min^−1^) up to 900 °C. The mass percentage of silica (% mass(SiO_2_)) was calculated from the residual mass at 900 °C. The EA of the hybrid materials was performed on an Interscience Flash EA 1112 series (Thermo Finnigan) instrument. The value of the mass percentage of OC (% mass (OC)) in the hybrid materials, %N, which in turn was used to calculate the DHBC weight percentage (% mass (DHBC)), %C, can be calculated according to the following equations:

[2] %N=% mass(OC)×% mass(N/OC)

[3] $$\begin{array}{c} \%\text{C}=\left(\%\text{ mass(OC)}\times \%\text{ mass(C/OC)}\right)\\ +\left(\%\text{ mass(DHBC)}\times \%\text{ mass(C/DHBC)}\right) \end{array}$$

where % mass (N/OC), % mass (C/OC) and % mass (C/DHBC), respectively stand for the mass content of N and C in OC and of C in the DHBC.

The degree of condensation (*D*) of the as-synthesized silica network was determined by ^29^Si MAS-NMR spectroscopy using a Varian VNMRS 300MHz spectrometer. The typical Q_4_, Q_3_ and Q_2_ signals appearing respectively at −110 ppm (SiO_2_), −100 ppm (SiO_3/2_) and −90 ppm (SiO(OH)_2_) were deconvoluted, and the areas were used for calculating *D*.

The textural properties of the materials were determined from the nitrogen sorption isotherms recorded at 77 K using a Micrometics Tristar 3000 apparatus. Prior to analysis, the samples were outgassed for 14 h under vacuum (0.08 mbar) at 250 °C for calcined materials and at 45 °C for hybrid materials. The surface area (*S*_BET_) was determined from the Brunauer–Emmett–Teller (BET) analysis in the relative pressure range corresponding to *p*/*p*^0^ < 0.4 and assuming a surface coverage of 13.5 Å^2^ per nitrogen molecule [31–32]. The mesopore volume was calculated using the α_S_ method; the diameter of cylindrical pores was determined from the adsorption branch using the nonlocal density functional theory (NLDFT) model [32] and the width of slit-shaped pores was estimated from the desorption branch using the method of Broekhoff and de Boer [33].

The structural properties were studied by small angle X-ray scattering (SAXS) and electron microscopy. SAXS measurements were performed in transmission mode on an in-house setup at the Laboratoire Charles Coulomb (Université Montpellier, France) using a high brightness, low power X-ray tube (λ = 1.5418 Å). All the intensities were corrected from transmission and empty capillary. Transmission electron microscopy (TEM) images were acquired on microtomed samples (slices of ≈70 nm thickness) with a JEOL 1200 EX II instrument operating at 120 kV. Material characterization by scanning electron microscopy (SEM) was done on a HITACHI S4800 (FEG-HR) apparatus operating at 5 kV.

## Results and Discussion

### Formation of polyion complex micelles

**Figure 1:**
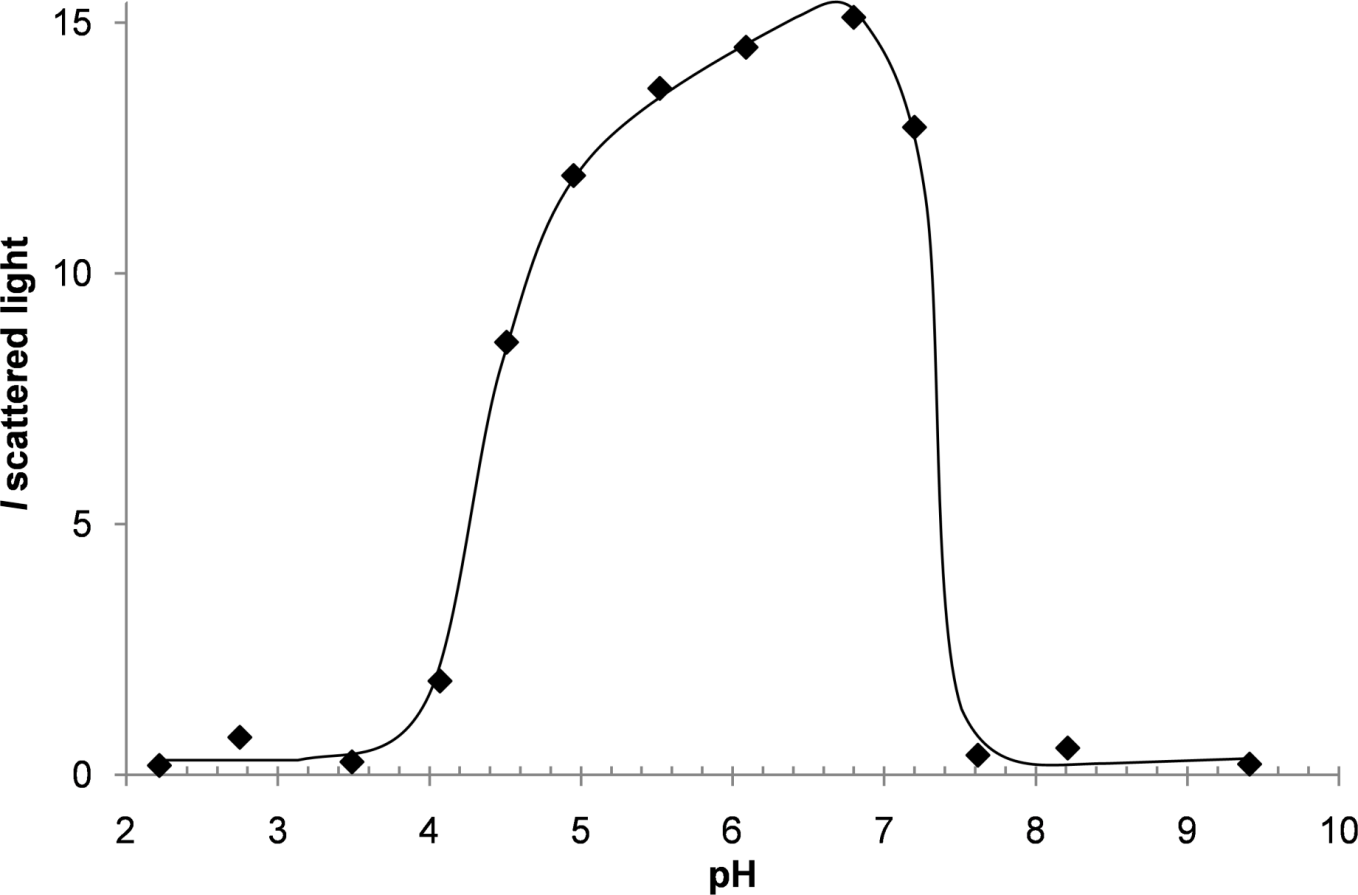
Variation of the scattered light intensity of PEO-*b*-PAA/OC mixtures ([AA] = 0.01 mol·L^−1^, N/AA = 0.8) as a function of pH.

The double hydrophilic block copolymer (DHBC) used in this study is a poly(ethylene oxide)-*b*-poly(acrylic acid) copolymer (PEO-*b*-PAA_,_ *M*_PEO_ = 5000 g·mol^−1^, *M*_PAA_ = 1420 g·mol^−1^) able to complex oligochitosan (OC) via electrostatic interactions in a suitable pH range where both the DHBC and OC are charged. The formation of polyion complex (PIC) micelles is evidenced by an increase of the scattered light intensity due to the formation of macromolecular assemblies. Figure 1 shows the variation of the scattered light intensity of mixtures of PEO-*b*-PAA and OC as a function of pH (2 < pH < 10). PEO-*b*-PAA/OC PIC micelles are obtained in the 4.5–7.2 pH range and are characterized by a mean hydrodynamic diameter (*D*_hv_) of ≈25 nm.

### Structure characterization of the calcined materials

**Figure 2:**
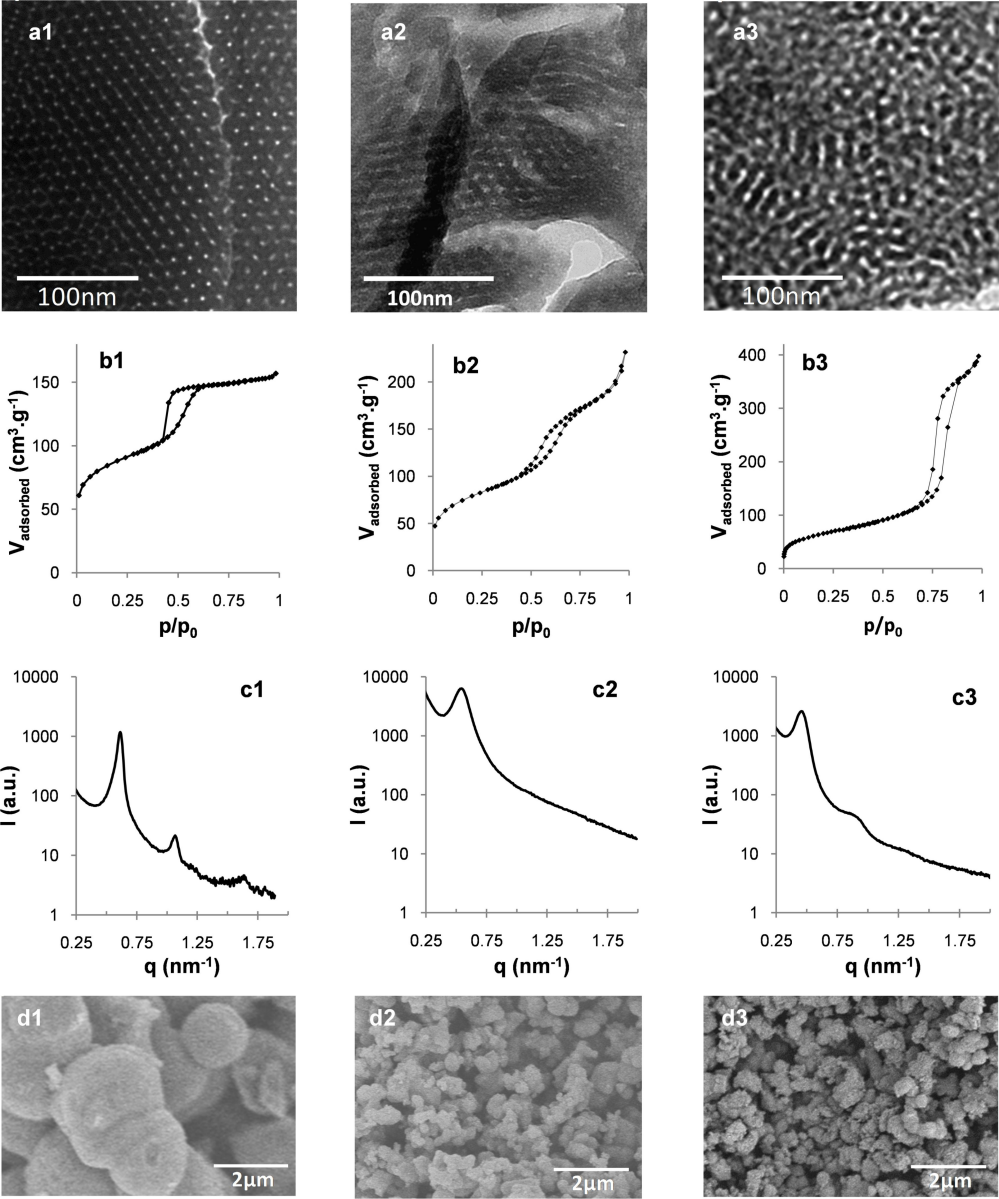
TEM micrographs (a), N_2_ adsorption/desorption isotherms (b), SAXS patterns (c) and SEM images (d) of the calcined materials synthesized with 3.9 wt % DHBC at pH 4.5 (d1), 6.5 (d2) and 7.9 (d3).

**Figure 3:**
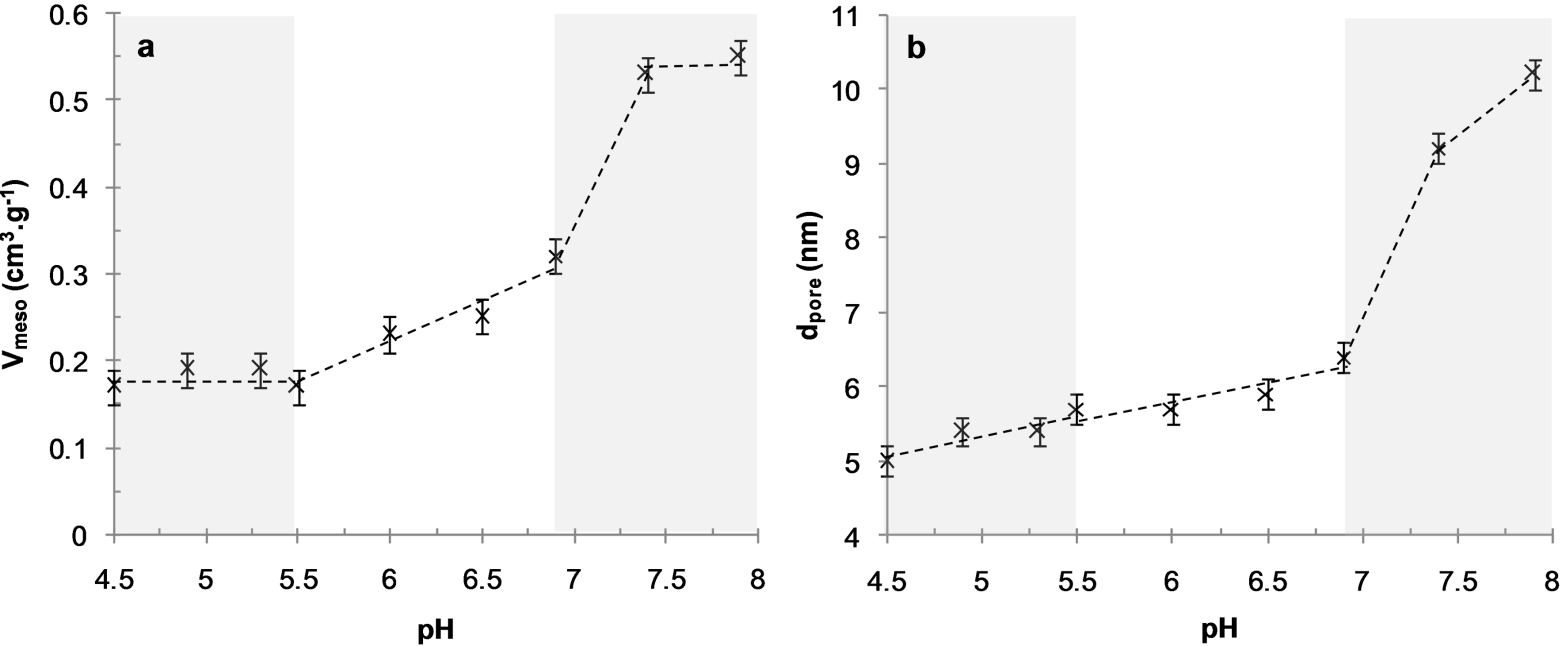
Mesopore volume (a) and pore diameter (b) of the calcined materials synthesized at 3.9 wt % DHBC at various values of pH.

Hybrid organic–inorganic materials were synthesized following a two-step approach. First, the reactants (TEOS, PEO-*b*-PAA and OC) were mixed together at low pH (pH 2) until a homogeneous solution of hydrolyzed silicic species, PEO-*b*-PAA and OC was obtained. Note that at pH 2 the silica structure-directing agent (SDA) constituted of PEO-*b*-PAA/OC PIC micelles is not formed (see Figure 1) due to the lack of favorable electrostatic interactions, whereas the organic–inorganic interface is thought to be formed by the N^0^/I^0^ pathway. Then the pH of the reaction medium was increased in order to promote both formation of PIC micelles and condensation of silica oligomers, inducing sudden macroscopic precipitation in less than 3 min. Two different mass concentrations of the reaction medium, expressed by the wt % of DHBC, were used. The 3.9 wt % concentration was aimed at evaluating the influence of the pH on the texture, structure and chemical composition of the obtained materials by carefully screening the 4–7.9 pH domain, whereas the 1.9 wt % concentration was used at only two strategic pH values (pH 4.5 and 6.5, see thereafter) with the aim of confirming the general effects observed at 3.9 wt %. After calcination (8 h at 550 °C), the materials synthesized under the various pH conditions presented an organization at the mesoscale as clearly evidenced on the TEM images of Figure 2a and Figure S1 in Supporting Information File 1, except in the case of pH 4 where a moderately porous silica material (*V*_meso_ = 0.11 g·cm^3^) with non-ordered small mesopores (*d*_pore_ = 3.9 nm) was obtained (see TEM in Figure S1, Supporting Information File 1). At pH 4, only very few PIC micelles were formed, as evidenced by the very low light scattered intensity (Figure 1); it is then obvious that the silicic species induced precipitation of a long range organized hybrid PIC-based mesostructure, which requires a sufficient amount of micelles, cannot occur. Interestingly, at the highest pH values (pH 7.4 and 7.9), ordered mesostructures were obtained. This could appear as a surprising result since these two pH values are outside of the micellization pH range (Figure 1), as determined in the absence of silica precursors. The formation of mesostructures at a pH above pH 7 can be understood by considering (1) the fact that the adjustment of pH at its final value is done by a progressive addition of a base solution up to the final pH, going inevitably through the micellization pH range where the structure-directing agent forms, and (2) the fact that the silica condensation rate regularly increases above pH 2 favoring the formation of the hybrid structure. These two features lead to the precipitation of the hybrid mesostructures at a pH well below the final targeted synthesis pH. These considerations will be further developed when discussing the silica material structuring below. At intermediate pH, but within the pH domain where micelles formed (Figure 1), the structure of the mesopores is 2D-hexagonal in the 4.5–5.5 pH range and a mixture of wormlike/lamellar at pH 5.5–6.9 (Figure S1, Supporting Information File 1). The mesopore volume increases from *V*_meso_ = 0.17 cm^3^·g^−1^ at pH 4.5 to 0.32 cm^3^·g^−1^ at pH 6.9, in accordance with the pore diameter change (*d*_pore_ = 5.0 nm at pH 4.5 and 6.4 nm at pH 6.9, see Table 1). Similarly, the lattice spacing value, *d*_0_, of the calcined materials shows a slight tendency to increase (Table 1). The *d*_pore_/*d*_0_ ratio is then almost constant (*d*_pore_/*d*_0_ ≈ 0.50), indicating that the mesostructure formation mechanism is similar within that pH range. Figure 3 helps to highlight the changes in the mesoporous volume (Figure 3a) and pore diameter (Figure 3b) observed upon synthesis of the materials on the whole pH range studied (4.5 ≤ pH < 7.9). It evidences three main pH domains (4.5 ≤ pH < 5.5, 5.5 ≤ pH ≤ 6.9 and 7.4 ≤ pH ≤ 7.9), for which the porous properties exhibit some common features together with the typical mesostructures of the materials illustrated on Figure 2a, which evolved from long-range ordered 2D-hexagonal (4.5 ≤ pH < 5.5) to a less-ordered worm-like/lamellar mixture (5.5 ≤ pH ≤ 6.9) to a short-range ordered honeycomb-like arrangement of cylindrical mesopores. For the sake of clarity, in the following, the influence of the synthesis pH on the material formation will be discussed by distinguishing these three main pH domains.

**Table 1:** Textural and structural properties of the calcined materials synthesized at 3.9 wt % DHBC: lattice parameter (*d*_0_), pore diameter (*d*_pore_), full width at half maximum of the pore size distribution (∆*d*_1/2_), mesoporous volume (*V*_meso_), external surface area (*S*_ext_) of the particles and particle size (*d*_particle_).

pH	4.5	4.9	5.3	5.5	6.0	6.5	6.9	7.4	7.9

*d*_0_ (nm)	10.2	11.1	10.6	11.6	11.9	11.5	11.3	13.5	13.8
*d*_pore_ (nm) ∆*d*_1/2_ (nm)	5.0 1.0	5.4 1.0	5.4 1.2	5.7 1.6	5.7 1.7	5.9^a^ 1.7	6.4^a^ 2.3	9.2 3.0	10.2 2.9
*V*_meso_ (cm^3^·g^−1^)	0.17	0.19	0.19	0.17	0.23	0.25	0.32	0.53	0.55
*S*_ext_ (m^2^·g^−1^)	13	15	27	39	73	125	123	124	130
*d*_particle_ (nm)	2300	1200	900	700	600	290	380	310	220

^a^mean pore diameter.

**Figure 4:**
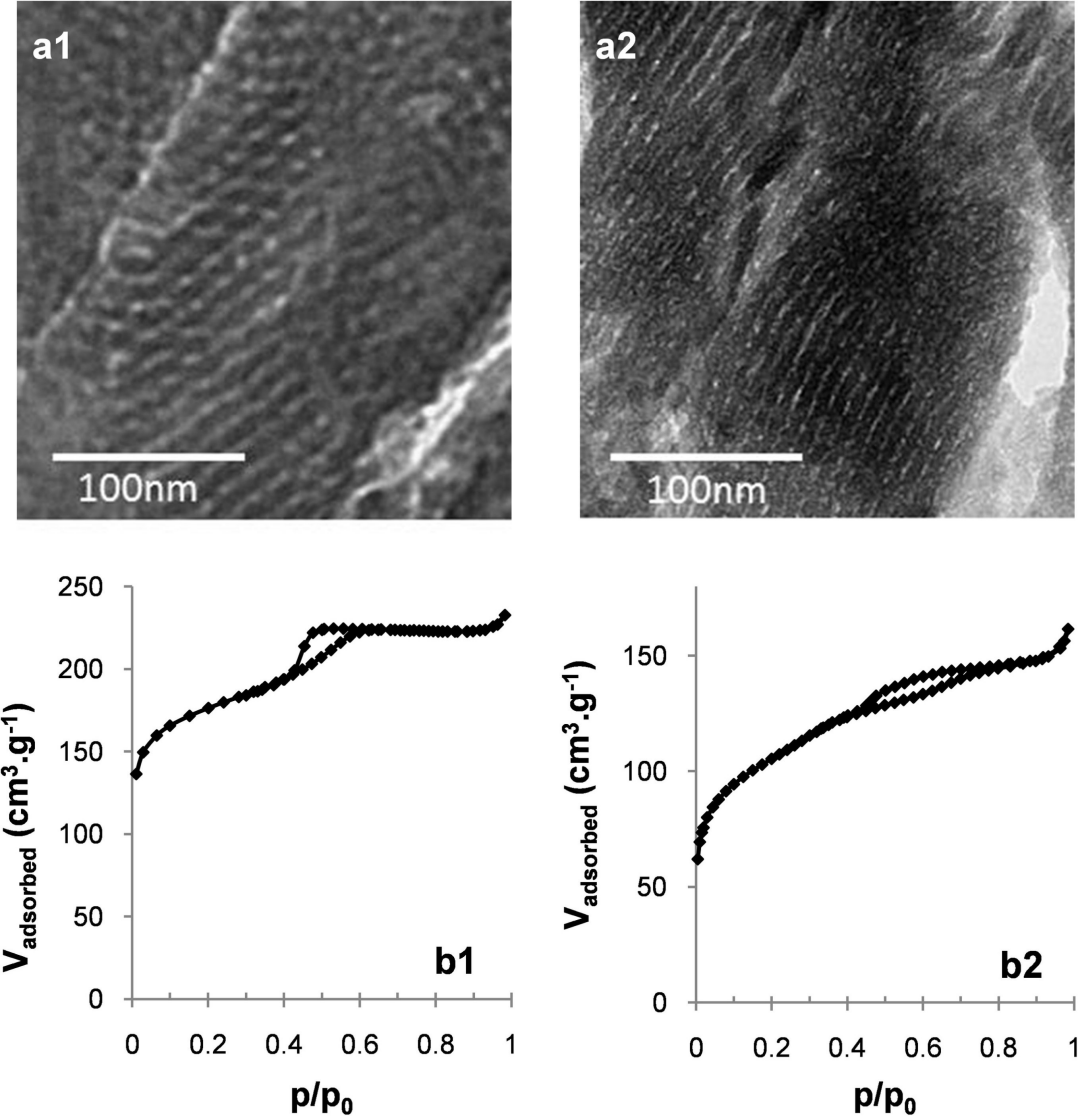
TEM micrographs (a) and N_2_ adsorption/desorption isotherms (b) of the calcined materials prepared at 1.9 wt % DHBC at pH 4.5 (b1) and 6.5 (b2).

In the most acidic domain (4.5 ≤ pH < 5.5), agglomerated spherical particles with a well-defined 2D hexagonal ordered mesostructure were obtained, as evidenced by TEM images (Figure 2a and Figure S1 in Supporting Information File 1). This mesostructure is confirmed by SAXS profiles (Figure 2c and Figure S2a in Supporting Information File 1), which exhibit up to four distinct scattering peaks whose relative positions respective to the first one appear at a ratio of 1:√3:√4:√7, corresponding to the (100), (110), (200) and (210) diffraction planes of long-range ordered hexagonally packed cylindrical structures (Table S1a, Supporting Information File 1). It should also be mentioned that the N_2_ sorption isotherms (Figure S3 in Supporting Information File 1) exhibited the typical type-IV shape of mesoporous materials with H1-like hysteresis loop (IUPAC classification [34]) showing capillary condensation at a relative pressure *p*/*p*_0_ ranging from 0.42 to 0.70. This indicates that the structural mesoporosity presents a cylindrical pore geometry with a high degree of pore size uniformity. This is confirmed by the narrow pore size distribution (PSD) calculated from the adsorption branch by the NLDFT method (Figure S3 in Supporting Information File 1). Within that pH range, the mean pore diameter (*d*_pore_) and mesopore volume (*V*_meso_) slightly increase with pH (Table 1). When the synthesis was performed at 1.9 wt % of DHBC and pH 4.5, a material with a mixed mesostructure consisting of lamellar domains coexisting with some 2D-hexagonal domains was obtained, as evidenced both by TEM images (Figure 4a) and SAXS profiles (Figure S2d in Supporting Information File 1), which exhibit two major diffraction peaks ascribed to the (100), and (200) planes of the lamellar/hexagonal structure and a weaker peak that could correspond to the (110) plane of the hexagonal structure. The nitrogen sorption isotherm (Figure 4b) exhibits a very low adsorption step, in good agreement with this mixture of mesostructures.

When further increasing the pH (5.5 ≤ pH ≤ 6.9), the well-ordered hexagonal mesostructure progressively evolved towards less-ordered structures containing domains with wormhole-like mesopores coexisting with domains of slit-shaped pores resembling short-range lamellar structure. Such lamellar domains appeared first (from pH 5.5) on the edge of the particles (see TEM images on Figure S1 in Supporting Information File 1). At pH 6.9, almost no hexagonal mesostructure was visible on TEM images. Moreover, the correlation peaks of the SAXS patterns broadened upon pH increase and their number decreased until a single peak was observed for materials synthesized at pH 6.9 (Table S1a and Figure S2 in Supporting Information File 1). This is in accordance with the loss of long range-ordered hexagonal structure, as observed in TEM images. In the sorption isotherms, the sharp adsorption step assigned to mesopore filling in the hexagonal materials becomes less pronounced, and the hysteresis loop extends on a wider partial pressure range, in agreement with the presence of lamellar domains coexisting with wormhole pore morphologies. The increase of pH from 5.5 to 6.9 resulted in a size pore increase with broadening PSD until pH 6 (Table 1), then to the appearance of a bimodal distribution with *d*_pore_ = 5.7 and 7.2 nm, with the fraction of the larger mesopores increasing with pH (from 12% to 48%). Accordingly, the mesopore volume increased within that pH range. Such observations reveal an increase of the mean radius of curvature of the mesostructure as a function of pH up to 6.9. Such an influence of the solution pH on the variation of mesostructure and mesopore size has also been reported with non-ionic surfactants/copolymers [11,20,35–36] and ionic cetyltrimethylammonium. [37] The up-curvature observed at *p*/*p*^0^ > 0.85 on the 5.5 ≤ pH ≤ 6.9 isotherms (Figure S3 in Supporting Information File 1) revealed an interparticle porosity, which is consistent with the small size of the silica particles as observed on SEM images (Figure S4 in Supporting Information File 1). As reported in Table 1, the mean particle size decreases from about 700 to 380 nm in the considered pH range. When the material synthesis was performed at a lower mass concentration (1.9 wt % of DHBC) and at pH 6.5, the material presented a well-defined lamellar mesostructure, which survived the removal of the SDA by calcination, as revealed by the TEM image of Figure 4a. The N_2_ sorption isotherm (Figure 4b) exhibited the classical H3-like hysteresis loop expected for such a mesostructure, with a mesoporous volume of 0.17 cm^3^·g^−1^. A pore thickness of 3 nm was calculated using the Broekhof and De Boer method from the relative pressure at which complete capillary condensation took place. [33].

The materials prepared at higher pH (pH 7.4 and 7.9) exhibited a relatively well-ordered pore arrangement, whose mesostructure was however difficult to identify (Figure 2a and Figure S1 in Supporting Information File 1). Nonetheless, some insights can be gathered from TEM, SAXS profiles and N_2_-adsorption/desorption data. Up to three scattering peaks with interplanar spacing ratios of 1:2:3 were revealed by SAXS for the material synthesized at pH 7.9 (Figure 2c and Table S1a in Supporting Information File 1), corresponding either to a lamellar structure or to a hexagonal one with some of the diffraction planes masked under broaden peaks. Let us add that the sorption isotherm did not exhibit the characteristic H3 hysteresis loops of mesoporous structures with slit-like pores, but rather the typical H1 type of cylindrical pore geometry. We thus propose that the mesostructures obtained at pH > 7 correspond to an hexagonal arrangement of cylindrical pores organized on very short distances that would account both for the TEM observation and SAXS data. Note that the pores of those materials are particularly large (*d*_pore_ = 9.2 and 10.2 nm at pH 7.4 and 7.9, respectively) compared to the mesopores obtained at lower pH. At pH 7.9, the pore diameter is about twice that of the material obtained at pH 4.5 (*d*_pore_ = 5.0 nm). This pore diameter increase is accompanied by a significant increase of the d-spacing value (*d*_0_ = 13.8 nm at pH 7.9, Table 1), compared to the almost constant value obtained between pH 4.5 and pH 6.9 (*d*_0_ = 11.3 ± 0.7 nm).

The pH increase also affected the size of the obtained primary spherical particles (see SEM images on Figure 2d and Figure S4 in Supporting Information File 1), which gradually decreased from several micrometers at low pH (2300 nm at pH 4.5) to a few hundred nanometers above pH 7 (220 nm at pH 7.9, Table 1). The external surface area of the particles, as measured by BET analysis, consistently increases within that pH range (Table 1). Similar particle size reduction upon pH increase was reported using polyethylene oxide based surfactants [38–39] as SDA of silica; it was ascribed to an increase of the polycondensation rate of silicic species with pH favoring fast nucleation of small flocs of surfactant and silica [40]. As emphasized by Berggren and Palmqvist [35], these small flocs further grow until reaching a final size that depends on the electrostatic stabilization provided by the pH-dependent negative charge of silica.

### Chemical composition of the hybrid materials and formation mechanism of mesostructures

**Figure 5:**
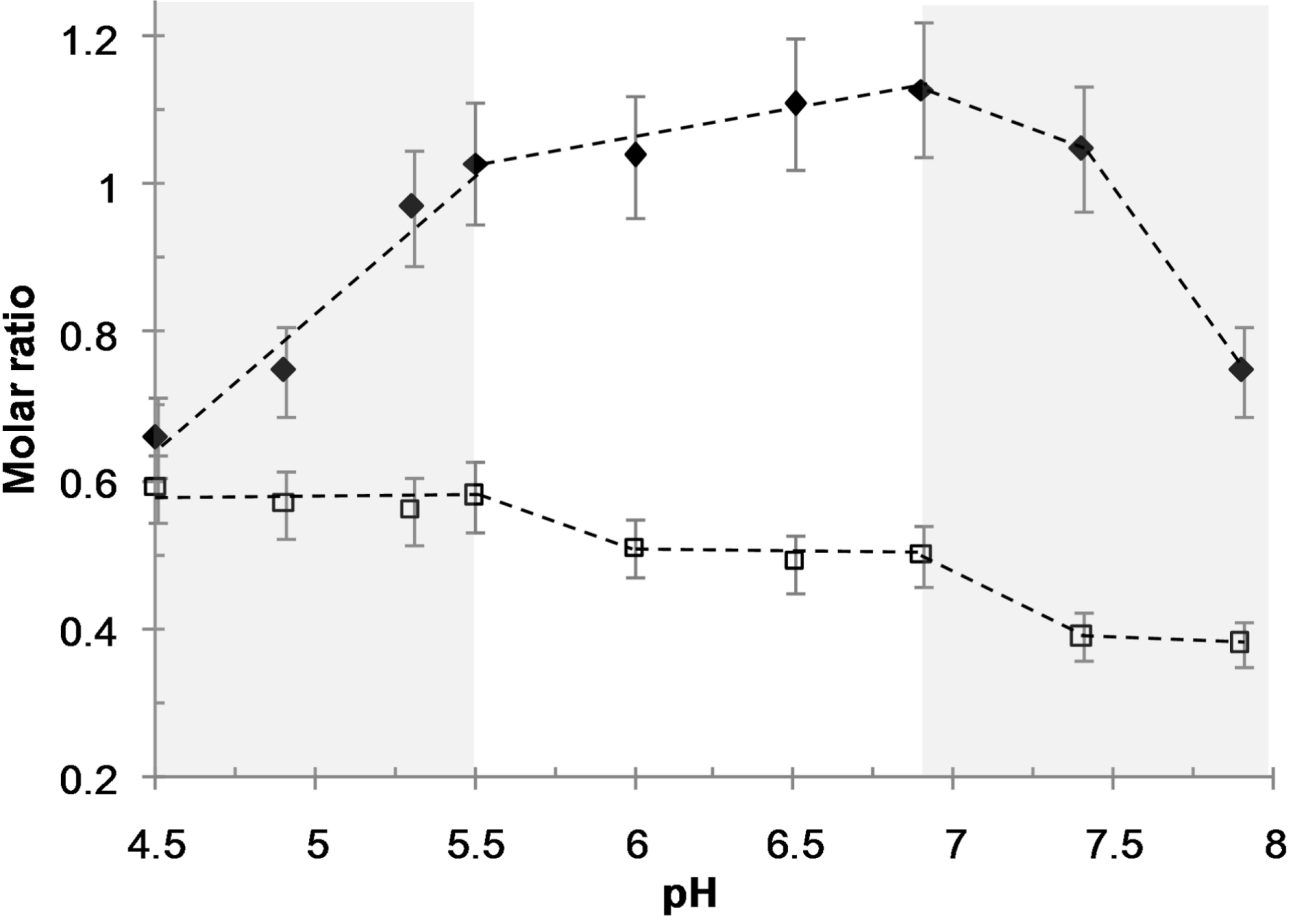
Molar ratio N/AA (filled triangles) and EO/Si (open squares) of the hybrid materials synthesized at 3.9 wt % DHBC as a function of pH.

The chemical composition of the as-synthesized hybrid materials was determined in order to understand how the pH of the reaction medium influences the interactions between the various constituents of the system and the subsequent mesostructures. The three main interactions to be considered are: the electrostatic interaction involved in the polyion complex formation between amine units (related to N atoms in OC) and acrylic acid units (AA in the DHBC), the hydrogen bond interaction ensuring the formation of the hybrid organic–inorganic interface between the ether oxygen (EO) of the PEO and the silica species (Si), and the self-condensation of silica species. The two molar ratios N/AA and EO/Si, respectively indicative of the extent of electrostatic complexation and hydrogen bonding, were determined from the chemical mass compositions of DHBC (wt % DHBC), oligochitosan (OC) (wt % OC) and silica (wt % SiO_2_) obtained by elemental analysis and thermogravimetric data after drying of the hybrid materials. The silica condensation degree (*D*) was quantified by ^29^Si MAS NMR spectroscopy. Table 2 gathers the data related to the compositions of the materials expressed in weight percentages and mg (organics) per gram of SiO_2_. Figure 5 shows the N/AA and EO/Si ratio variations in the materials as a function of pH.

**Table 2:** Chemical composition of the as-synthesized hybrid materials synthesized at 3.9 wt % DHBC.

pH	4.5	4.9	5.3	5.5	6.0	6.5	6.9	7.4	7.9

wt % SiO_2_	51.8	53.1	50.4	49.0	52.0	53.1	51.8	58.5	60.4
DHBC (mg·g_SiO2_^−1^)	553	536	528	545	480	456	468	370	361
OC (mg·g_SiO2_^−1^)	211	233	299	325	290	294	306	225	157
*D* (%)	86	86	87	88	87	88	88	91	90

For all pH values of the reaction medium, the materials present a high silica content (SiO_2_ wt % > 50%, associated with high silica yield above 80%) and a highly condensed inorganic network (*D* > 86%). As the pH increased, the degree of condensation rose (from *D* = 86% at pH 4.5 to 91.5% at pH 7.4), in agreement with the pH-dependent condensation rate of silica. The amount of PEO-*b*-PAA incorporated in the materials was relatively high and the variation depends on the pH domain already discussed above. At 4.5 ≤ pH < 5.5, the DHBC content was almost constant (average 540 ± 15 mg·g_SiO2_^−1^), and it slightly decreased between pH 5.5 and 6.9 (468 mg·g_SiO2_^−1^ at pH 6.9) and decreased to a higher extent above pH 7.4 where it reached a plateau (average 365 ± 5 mg·g_SiO2_^−1^ for pH ≥ 7.4). The OC content in the materials was much smaller than the DHBC amounts. The OC content relative to silica varies non-monotonically with pH: it increases from pH 4.5 to pH 5.3 (from 211 to 299 mg·g_SiO2_^−1^, respectively), and remained constant up to pH 6.9, and decreased strongly in the highest pH range to reach 157 mg·g_SiO2_^−1^ at pH = 7.9. The changes of N/AA and EO/Si ratios reported on Figure 5 reflect those organic content variations.

In the most acidic domain range (4.5 ≤ pH < 5.5), in which 2D hexagonal mesostructures were obtained, the N/AA increase (from about 0.65 up to a value of ≈1) accounts for the rise of the ionization degree of the acrylate functions expected for this weak polyacid (p*K*a_AA_ 4.8). The number of negative charges on the PAA increases with pH what favors the interaction with OC in the formation of polyion complex (see Figure 1), and allows more OC to be integrated in the material. This is consistent with the increase of the pore diameter of the calcined materials from 5 to 5.7 nm (Table 1 and Figure 3b). The EO/Si ratio is almost constant (EO/Si ≈0.6), which highlights the favorable hydrogen bond interactions between the ethylene oxide groups of PEO blocks and hydrolyzed silicic species (Si–OH) in this pH range.

At an intermediate pH (5.5 < pH ≤ 6.9), the N/AA increase may reflect not only the decrease of the charge density of oligochitosan (p*K*a_OC_ 6.7) but also the increase of the PAA charge density, leading to an increase of the OC content relative to the DHBC in order to compensate the PAA charge in the PIC nanophase. Let us note that the fact that the amount of OC integrated in the material (OC/SiO_2_) remained constant within that pH range could be due either to the formation of a polyion complex richer in OC or to the development of favorable interactions between negatively charged silica species and OC species, which could also favor OC incorporation. Even if this last interaction cannot be totally ruled out, the increase of the mean pore diameter in the calcined materials synthesized at 3.9 wt % of DHBC (Table 1) argues in favor of an OC-richer polyion complex. In such conditions, a looser polyion complex rich in OC is expected to form, in accordance with the increased mesopore volume. The EO/Si decrease reflects the weaker hydrogen bond interactions between EO and silica species, which are expected due to the pH-dependence of the charge density of silica species [40]. This decreased EO/SiOH interaction with pH is in good accordance with the well-admitted mechanism of formation of Pluronic-templated mesostructured silica [5,41–43], in which the primary step for a good mesostructure to be obtained is entropy driven by hydrogen bonding between the silica oligomers and PEO chains [5]. Variations in the respective sizes of the two nanodomains of the system, (1) the polyion electrostatic complex core and (2) the PEO/inorganic corona, as a function of pH, lead to changes of the interfacial curvature of the system that controls the mesostructure of the material. At 3.9 wt % of DHBC, the well-ordered 2D-hexagonal mesostructure of the pH 4.5–5.3 domain, presenting purely cylindrical mesopores, thus evolves towards a mixture of less-ordered worm-like/lamellar structures with larger pore diameter and the subsequent appearance of a bimodal distribution of mesopores at higher pH. It is noted that from pH 4.5 to 6.9, the pH increase leads to a decrease of the mean curvature of the mesostructure, as revealed by the increase of the pore diameter and/or the tendency to form domains of lamellar structures at the expense of 2D hexagonal structure domains. Let us add that these variations can be related to the increase in OC content in the PIC nanodomains.

At the highest pH values (pH 7.4 and 7.9), the N/AA ratio sharply drops whereas EO/Si further decreases. The low value of N/AA is quite surprising since the charge density of OC decreases strongly above pH 6.5, and then even higher values of N/AA could be expected as a result of the necessary charge compensation in the PIC nanophase. Let us note that the low value of N/AA is associated with a significant porosity of the as-synthesized hybrid materials (see Figure S5 in Supporting Information File 1). Large mesopore volumes (*V*_meso_ = 0.39 and 0.29 cm^3^·g^−1^ at pH 7.4 and 7.9, respectively) and large pore diameters (*d*_pore_ = 9.1 and 10.2 nm at pH 7.4 and 7.9, respectively) in the hybrid materials were obtained; they are close to the ones obtained on the calcined materials (Table 1). Those combined observations suggest that part of the oligochitosan molecules would be eluted from the material while the reaction medium is maintained for hours at pH above 7, as it can be expected when considering the micellization pH range (Figure 1) The occurrence of mesoporosity in the dried hybrid materials is currently under investigation and will be discussed further in a forthcoming paper. The very low value of the EO/Si ratio indicates a decay of the extent of the SiOH/EO hydrogen bond interaction compared to the syntheses performed at lower pH. Mesopore volumes (above 0.5 cm^3^/g) and pore diameters (about 10 nm) observed on the calcined materials (Figure 3 and Table 1) are much higher than at pH below 7, suggesting that the PEO block was not trapped into silica walls of the materials but rather acted as a porogen agent contributing to the mesopore volume once the materials were calcined. Such an occurrence is reminiscent of the size increase of the structural mesopores of SBA-15 materials observed at temperature of synthesis higher than 80 °C, which reduces the solvation of the PEO chains and thus weakens the interaction between PEO and silica [44]. A similar effect was observed with the present PIC structure directing agent when performing a material synthesis at pH 6.5 at 80 °C for 24 hours: the mesopore diameter of the calcined material increased from 5.9 to 12 nm and the mesopore volume from 0.25 to 0.95 cm^3^·g^−1^ (Figure S6 in Supporting Information File 1). The material contained a lower amount of DHBC (EO/Si 0.4 and 315 mg·g_SiO2_^−1^ instead of EO/Si 0.5 and 456 mg·g_SiO2_^−1^ at 30 °C) as it is the case for the synthesis performed at high pH, and it is also slightly poorer in OC (N/AA 1.07 and 239 mg/g_SiO2_^−1^ instead of N/AA 1.1 and 294 mg/g_SiO2_^−1^ at 30 °C). Interestingly, the mesostructure observed on the TEM image (Figure S7 in Supporting Information File 1) of such a temperature-treated sample is similar to the short-range ordered cylindrical mesopores presenting honeycomb-like arrangement of the materials synthesized at pH 7.4 and 7.9, thus supporting the role of the weaker interaction between PEO and silica at those pH. It is worth noting that the involvement of mediating cations ensuring the interface neutrality through a N^0^Na^+^I^−^ pathway (in our case Na^+^ coming from the sodium hydroxide solution used to adjust the pH) that has been proposed in some studies [4] does not hold in this high pH material synthesis, since the Na/Si molar ratio obtained from EDX measurements was too low (Na/Si ≈0.04) to support such an assembly.

**Figure 6:**
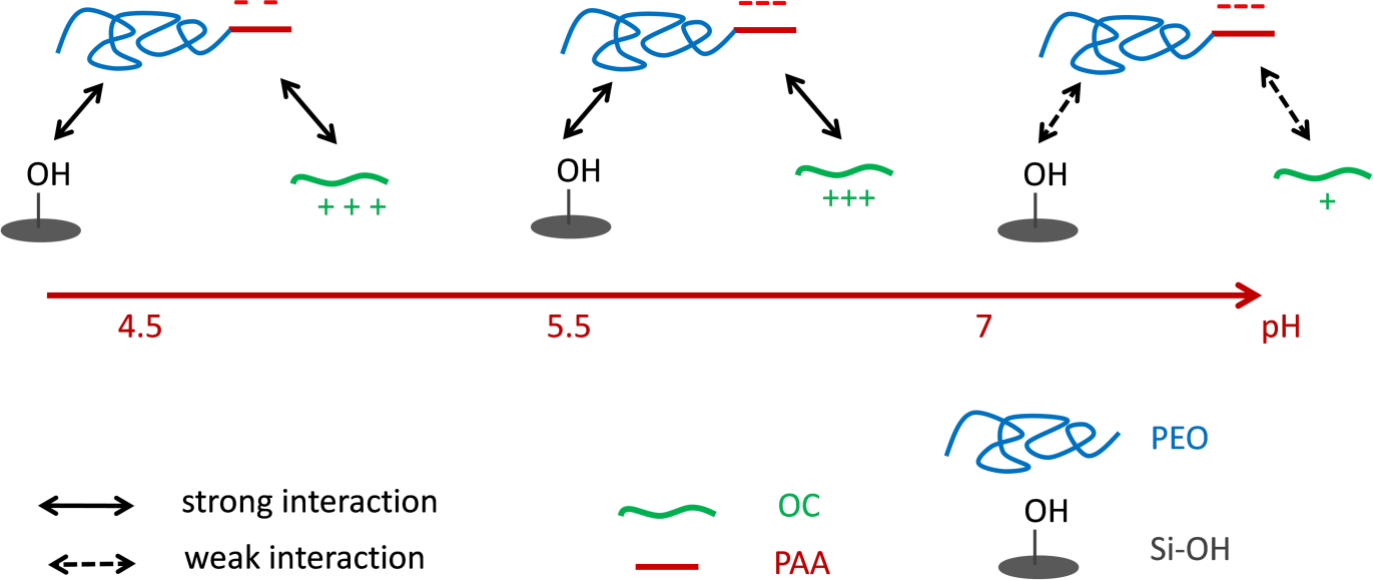
Schematic representation of the interactions between silicic species and PEO, OC and PAA entities as a function of pH.

The pH-dependent mesoproperties of the PEO-*b*-PAA/OC PIC structuring agent can be depicted by defining an induced amphiphilic unit that determines the nature of the mesophase. This amphiphilic unit can be described as a ternary system constituted of OC, PEO-*b*-PAA and silica species. The core of the amphiphilic system is constituted by a polyion electrostatic complex of OC/PAA and the corona by the H-bonded assembly of PEO block and silica species. As the pH of the synthesis medium is increased (from pH 4.5 to 6.5), the extent of the electrostatic interactions increases and the hydrogen bond interaction with silica species weakens, whereas the condensation rate of silica increases. Figure 6 schematically depicts the different interactions involved in the formation of the hybrid precipitate, and how they vary with pH, allowing us to tune the relative sizes of the electrostatic complex core and the PEO/inorganic corona of the amphiphilic unit. The pH sensitivity of the various interactions provided by this peculiar amphiphilic system allows tuning the mesostructure of the material from hexagonal to worm-like/lamellar simply by varying the pH of the reaction medium at 3.9 wt % of DHBC. At a lower DHBC concentration (1.9 wt %), the mesostructure transformed from lamellar/hexagonal at pH 4.5 to purely lamellar at pH 6.5. Interestingly, the hybrid materials prepared in more dilute conditions exhibited N/AA (0.62 and 1.24 at pH 4.5 and 6.5, respectively) and EO/Si (0.56 and 0.48 at pH 4.5 and 6.5, respectively) ratios similar to those obtained at 3.9 wt % whereas the mesostructures were found to be rather different from those obtained at 3.9 wt %. Several factors may be involved in the difference in mesostructures between the two concentrations of the reaction medium (hexagonal vs lamellar/hexagonal mixture at pH 4.5 and short-range ordered worm-like/lamellar mixture vs purely lamellar at pH 6.5): the different silica condensation rates, the different quantities of ethanol released upon TEOS hydrolysis and their influence on the polymer solubility, and the amounts of water contained in both the hydrophilic PEO shell and the PIC core, which may swell differently the two different compartments.

## Conclusion

In conclusion, mesoporous materials of various structures including 2D hexagonal, worm-like, and lamellar structures were formed by using polyion complex micelles as structure-directing agents under different pH conditions of material synthesis. As a weak polyacid-containing DHBC and a weak polybase were chosen as constituents of the polyion complexes, micelle formation occurs on a restricted pH domain between about 4.5 and 7. The silica framework is obtained by condensation of silicic species, which are formed by hydrolysis of the silicon alkoxide at pH 2. Due to the pH dependence of the PIC micelle properties, of the silica condensation rate, and of the PEO-silanol interactions, the variation of the pH of the reaction medium from 4.5 to about 8 led to considerable changes in the structural, textural and compositional properties of the materials.

After hydrolysis of TEOS in the presence of the polymers, the increase of the pH of the aqueous mixture between 4.5 and 7.9 leads to the formation of a macroscopic hybrid organic–inorganic precipitate. When the materials were synthesized in the pH 4.5–5.5 range, long-range ordered 2D hexagonal structures exhibiting pore diameters which regularly increase with pH were obtained. In the pH 5.7–6.5 range, a mixture of short-range ordered worm-like and lamellar structures was obtained; it appears to be a pure long-range ordered lamellar phase when the reaction medium was twice less concentrated. When the pH of the material synthesis exceeds pH 7, cylindrical pore morphologies were obtained, exhibiting some short-range ordered 2D hexagonal arrangement. In the pH range 4.5–6.9, pore diameters were shown to increase progressively with pH up to 5.7 nm, whereas they dramatically increase above pH 7 exceeding 10 nm. The variations of the structural and porous properties of the materials were shown to be related to variations of the compositions. Mesostructures with larger pore diameters or with a higher radius of curvature were obtained in the pH range 4.5–6.9, in relation with increased content of the polybase (oligochitosan) in the PIC nanodomain (increased ratio between amine and acrylate functions).

In summary, the pH of the reaction medium appears to be a key parameter in the determination of the structural and textural characteristics of mesoporous materials whose synthesis is directed by polyion complex micelles. This is due to the variation of three essential properties as a function of pH: the extent of the electrostatic bonding between the weak polyelectrolytes, the extent of the hydrogen bond interaction between silanol and PEO ether groups, and finally, the silica condensation rate. As a complementary investigation, the influence of other synthesis parameters, which were identified to play a role in the structure determination, including the concentration of the reaction medium and the temperature, is currently under study.

## Supporting Information

File 1 Additional experimental data.
