# Supplementary material for: pH-mediated control over the mesostructure of ordered mesoporous materials templated by polyion complex micelles
Source: Beilstein J Nanotechnol. 2019 Jan 11;10:144–56. doi: 10.3762/bjnano.10.14 (PMC6334808; doi:10.3762/bjnano.10.14)
Supplement: File 1 — Additional experimental data. [file Beilstein_J_Nanotechnol-10-144-s001.pdf]

BEILSTEIN JOURNAL OF NANOTECHNOLOGY

Supporting Information

for

pH-mediated control over the mesostructure of ordered mesoporous materials templated by polyion complex micelles

Emilie Molina, Mélody Mathonnat, Jason Richard, Patrick Lacroix-Desmazes, Martin In, Philippe Dieudonné, Thomas Cacciaguerra, Corine Gérardin and Nathalie Marcotte

Beilstein J. Nanotechnol. **2019,** *10,* 144–156. doi:10.3762/bjnano.10.14

Additional experimental data

License and Terms: This is a supporting information file under the terms of the Creative Commons Attribution License (http://creativecommons.org/licenses/by/4.0). Please note that the reuse, redistribution and reproduction in particular requires that the authors and source are credited.
The license is subject to the *Beilstein Journal of Nanotechnology* terms and conditions: (https://www.beilstein-journals.org/bjnano)

1. Further characterization of the mesoporous materials after calcination

1.1. Transmission Electron Microscopy (TEM)

The TEM images of the calcined materials obtained at a 3.9 wt% of DHBC concentration with a N/AA ratio of 0.8 reveal different mesostructures as a function of the pH of the reaction medium (Figure S1). Figure S1 presents some supplementary results on a wide range of pH from pH 4 to 7.9.

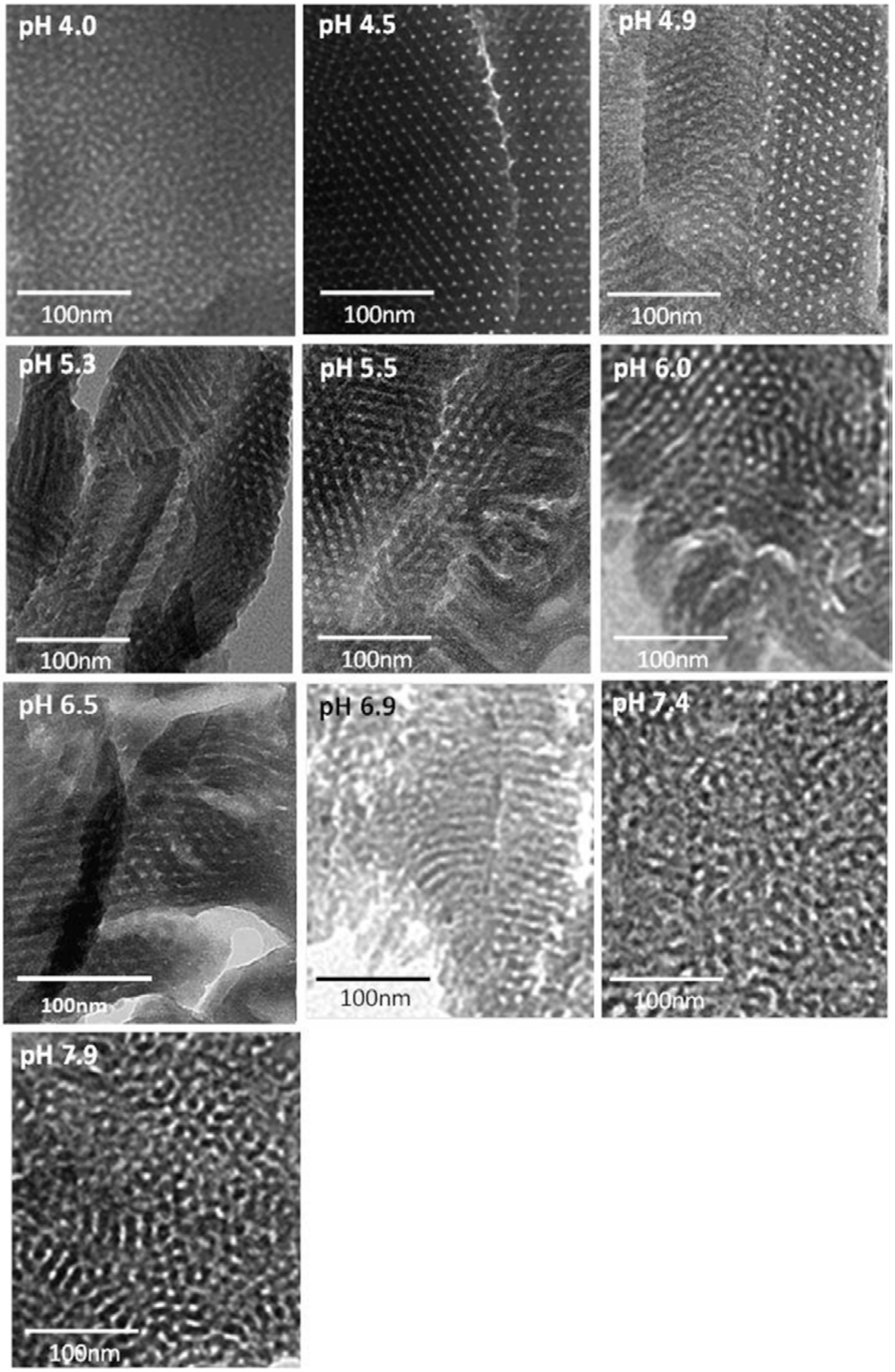

Figure S1: Transmission electron microscopy images of calcined materials structured by PEO-*b*-PAA/OC at 3.9 wt % DHBC at the following pH values 4.0, 4.5, 4.9, 5.3, 5.5, 6.0, 6.5, 6.9, 7.4, 7.9.

1.2 Small Angle X-ray Scattering

SAXS profiles (Figure S2) of the calcined materials obtained using 3.9 wt% of DHBC with N/AA = 0.8 reveal the mesostructures of the materials. The data related to the SAXS analyses are given in Table S1.

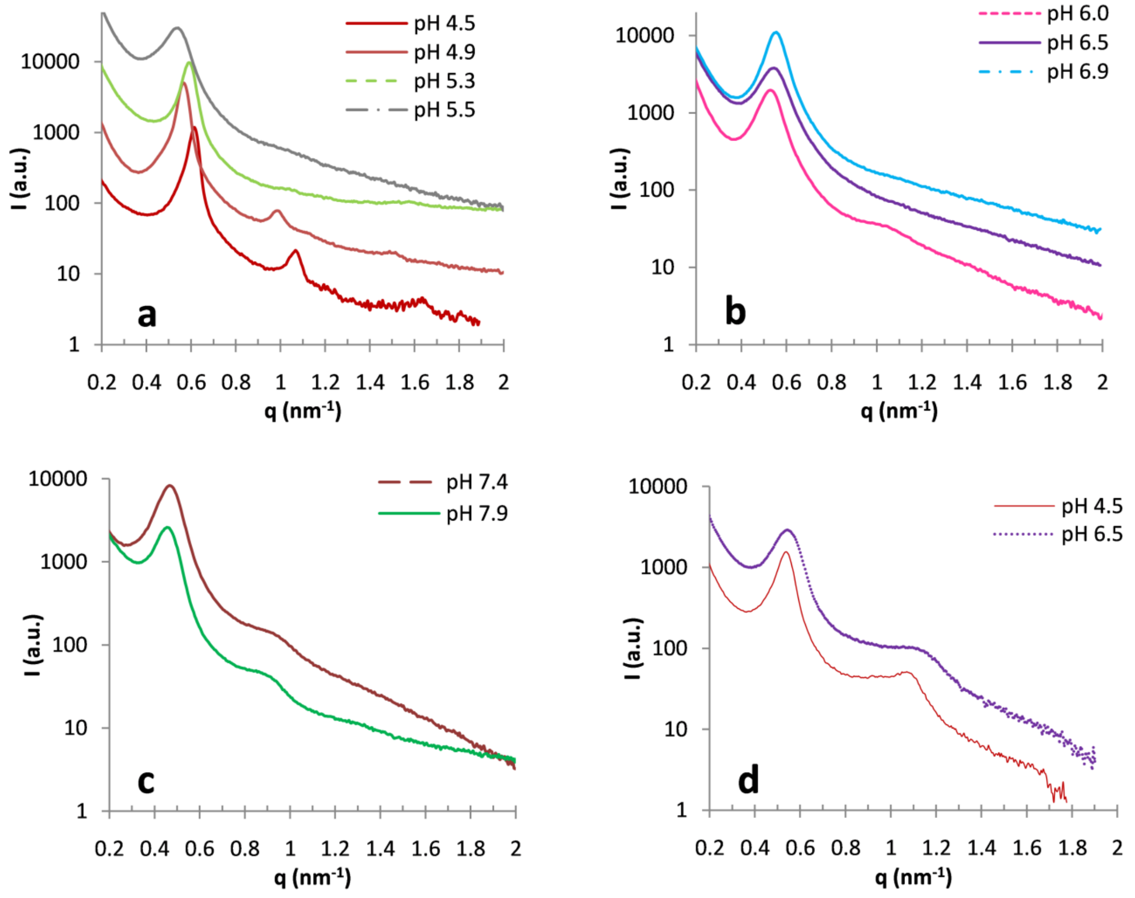

Figure S2: SAXS patterns of calcined materials structured by PEO-*b*-PAA/OC at 3.9 wt % DHBC (a,b,c) and 1.9 wt % DHBC (d).

Table S1: SAXS results of calcined materials structured by PEO-*b*-PAA/OC at 3.9 wt % (a) and 1.9 wt % (b).

(a)

**pH**	**4.0**	**4.5**	**4.9**	**5.3**	**5.5**	**6.0**	**6.5**	**6.9**	**7.4**	**7.9**
**d_0 (nm)**	11.1	10.2	11.1	10.6	11.6	11.9	11.5	11.3	13.5	13.8
**d_0/d_1**	-	1.74	1.75	1.74	-	-	-	-	-	-
**d_0/d_2**	-	2.02	1.99	1.99	1.90	1.98	2.05	-	2.0	1.99
**d_0/d_3**		2.67	2.68	2.65	2.60	2.75	-	-	3.1	2.92
**d_0/d_4**			2.98							

(b)

**pH**	**4.5**	**6.5**
**d_0 (nm)**	11.0	11.5
**d_0/d_1**	(1.73)	1.94
**d_0/d_2**	1.99	-

1.3. Nitrogen sorption

Nitrogen sorption isotherms and pore size distributions from NLDFT method of calcined materials obtained using PIC micelles with 3.9 wt% of DHBC and N/AA = 0.8 allow giving information on the textural properties (Figure S3).

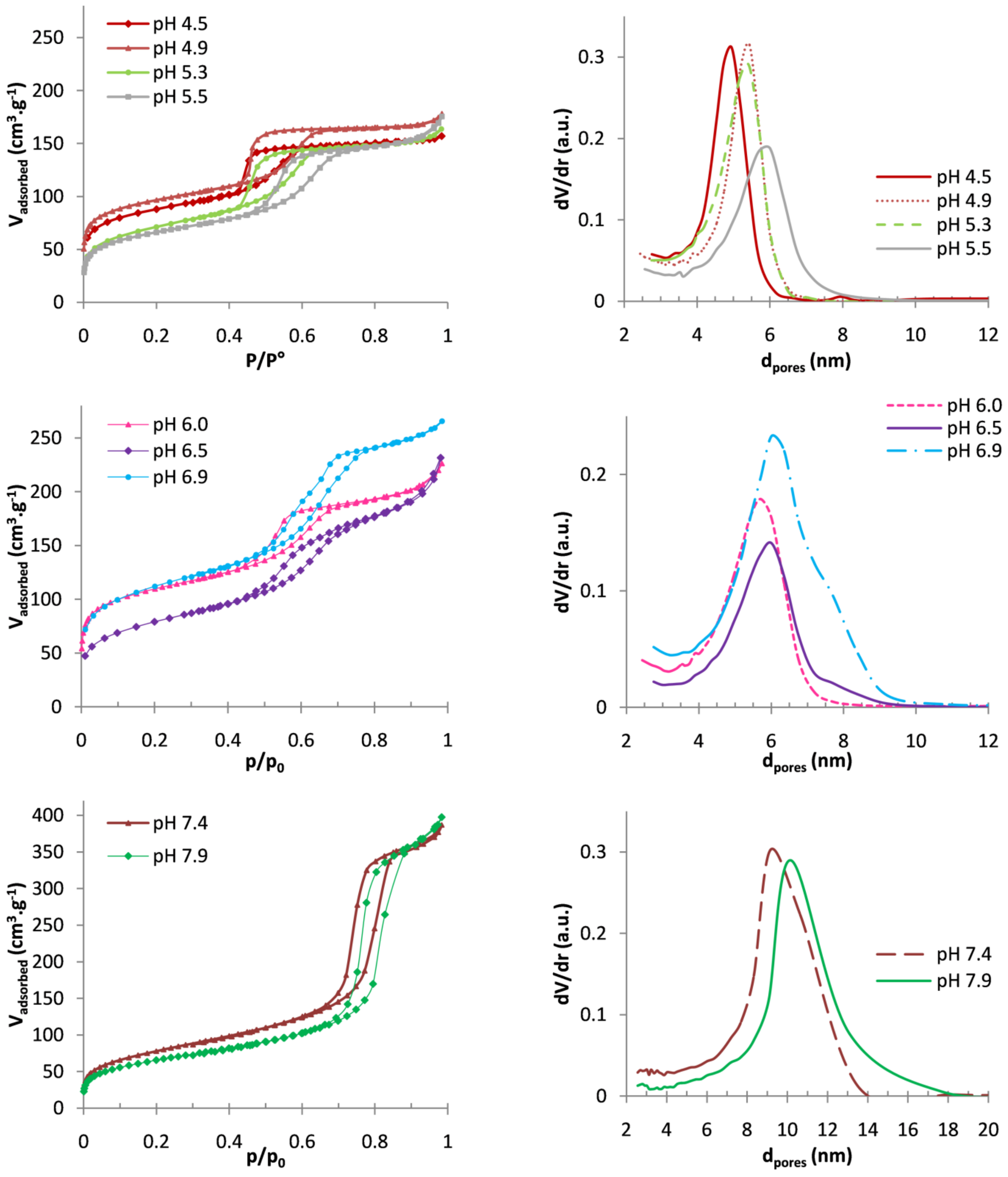

Figure S3: Nitrogen sorption isotherms (on the left) and pore size distributions from NLDFT method (on the right) of materials synthesized by PEO-*b*-PAA/OC at 3.9 wt % of DHBC.

1.4. Scanning Electron Microscopy (SEM)

SEM images of the calcined materials obtained using DHBC at 3.9 wt% and OC (N/AA = 0.8) at various pH values are shown on Figure S4. The size of the primary particles regularly decreases with the pH value.

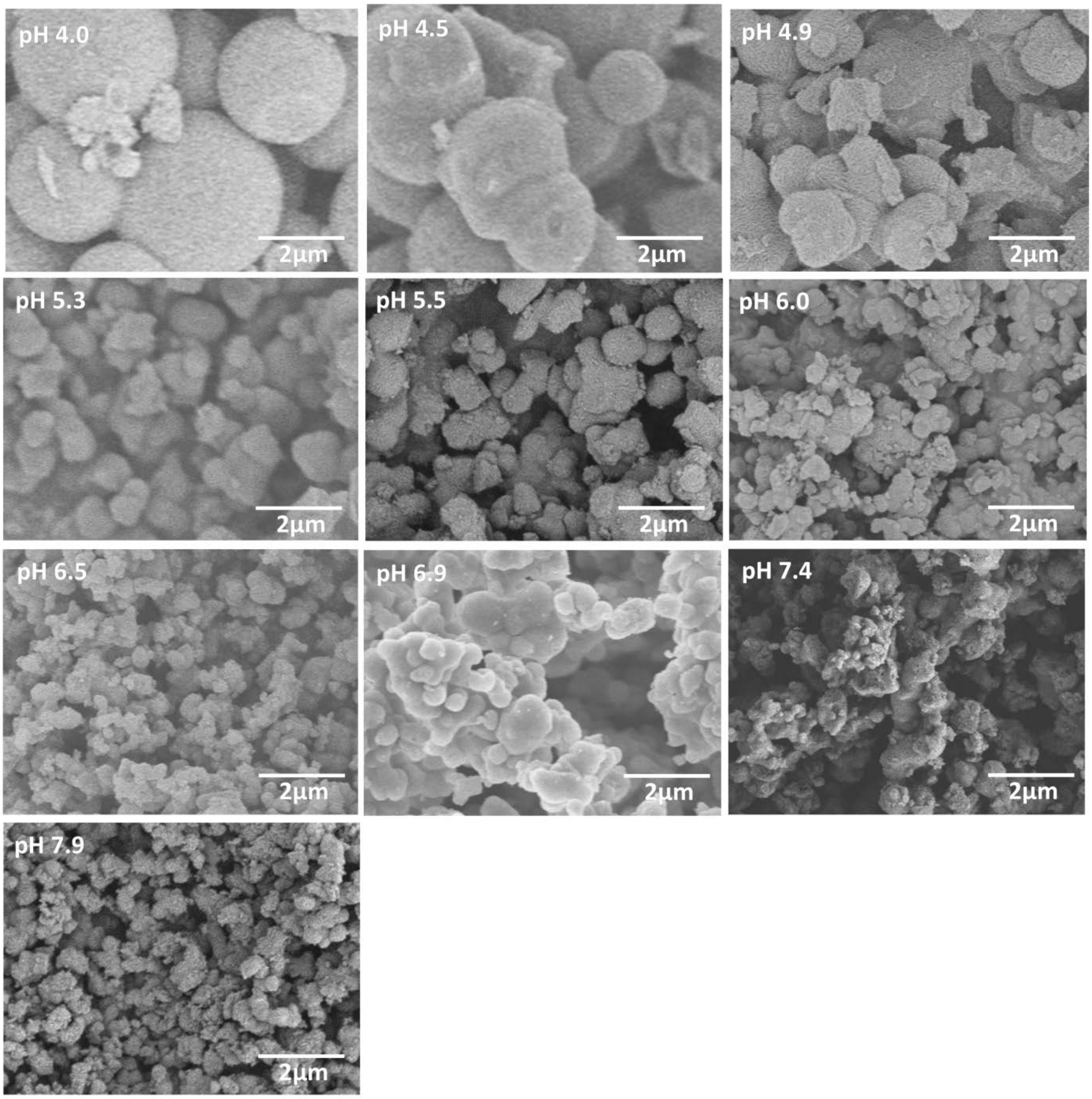

Figure S4: Scanning Electron Microscopy images of the calcined materials structured by PEO-*b*-PAA/OC at 3.9 wt % DHBC at pH 4.0, 4.5, 4.9, 5.3, 5.5, 6.0, 6.5, 6.9, 7.4, 7.9.

2. **Textural characterization of hybrid materials synthesized at pH above 7.0**

At the highest pH values (pH = 7.4 and 7.9), the as-synthesized hybrid materials exhibited porous properties with large mesopore volumes and wide pore diameters (Figure S5).

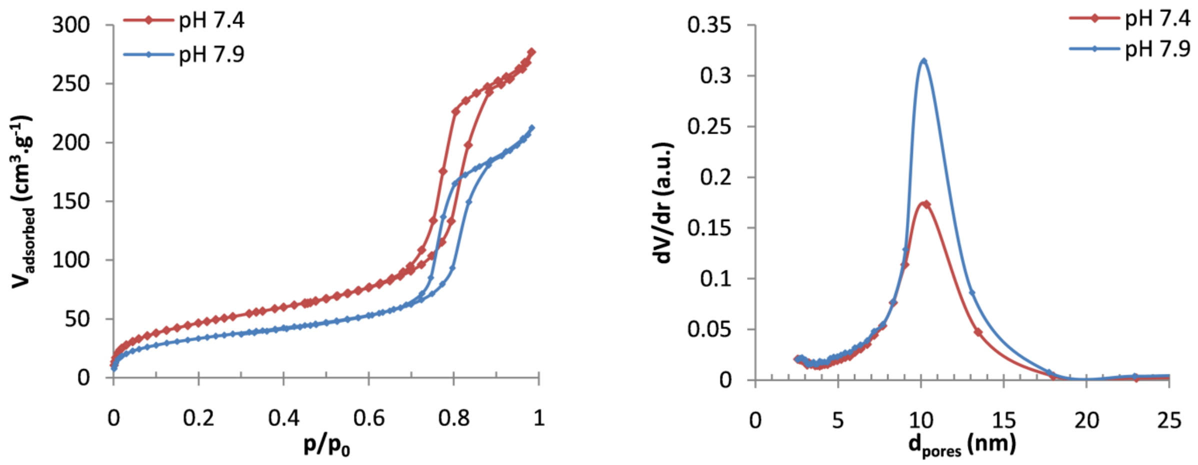

Figure S5. Nitrogen sorption isotherms and pore size distributions from the NLDFT method of the as-synthesized hybrid materials structured with DHBC at 3.9 wt % at pH = 7.4 and 7.9.

3. **Effect on the mesostructure of a higher temperature (80°C) after pH adjustment**

A material synthesis was performed at pH 6.5 at 80°C: the temperature was increased after the pH adjustment. The data show that the mesopore size of the calcined material increased from 5.9 to 12 nm and the mesopore volume from 0.25 to 0.95 $cm^3.g^{-1}$ when the temperature was changed from 30 to 80°C. Moreover, the material synthesized at 80°C is poorer in DHBC (315 $mg.g_{SiO2}^{-1}$ instead of 456 $mg.g_{SiO2}^{-1}$ at 30°C) as it is the case for the synthesis performed at high pH, and also slightly poorer in OC (239 mg/g_{SiO2}^{-1} instead of 294 mg/g_{SiO2}^{-1} at 30°C). These observations suggest that the PEO block was not trapped into silica walls of the materials but rather acted as a porogenic agent contributing to the mesopore volume once the material was calcined.

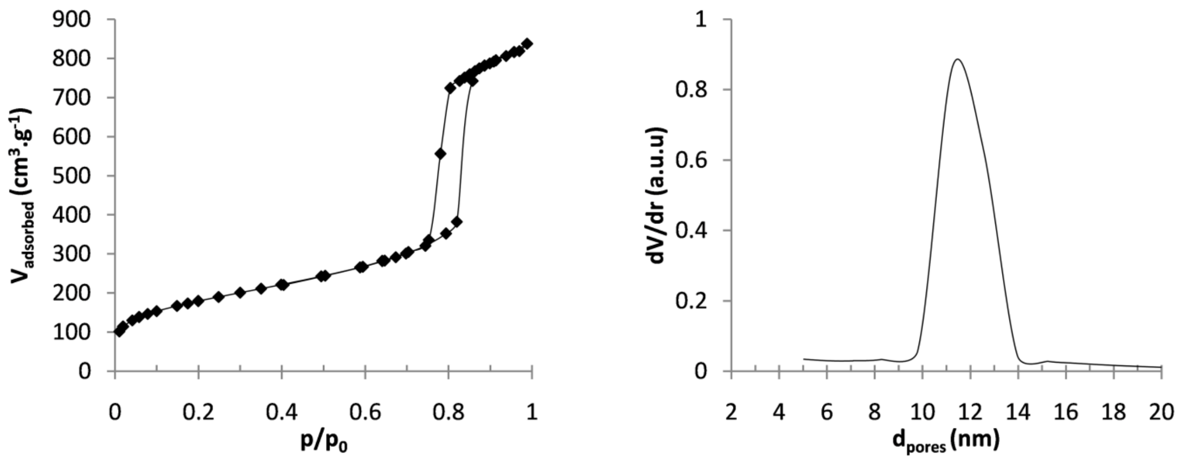

Figure S6: Nitrogen sorption isotherms (on the left) and pore size distribution from the NLDFT method (on the right) of material synthesized using PEO-*b*-PAA/OC at 3.9 wt % DHBC, pH 6.5 and with a temperature increase up to 80°C after the pH adjustment.

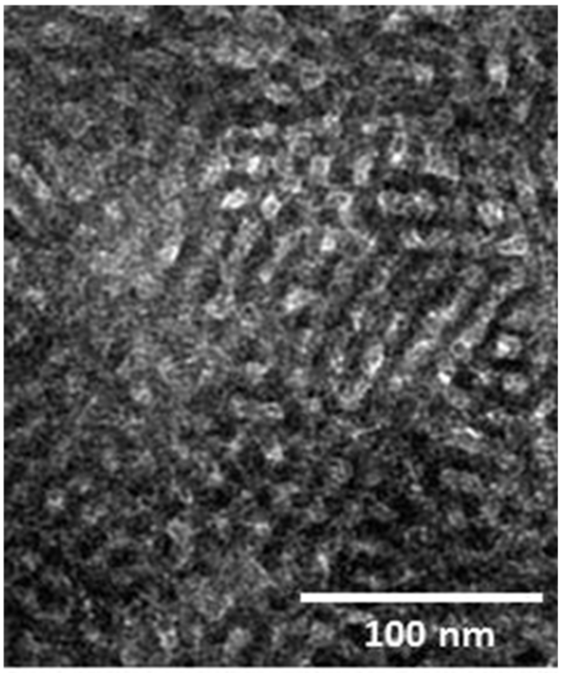

Figure S7: Transmission electron microscopy image of the calcined material synthesized using PEO-*b*-PAA/OC at 3.9 wt % DHBC, pH 6.5 and with a temperature increase up to 80°C after the pH adjustment.
